# Platelets and Intravascular Immunity: Guardians of the Vascular Space During Bloodstream Infections and Sepsis

**DOI:** 10.3389/fimmu.2019.02400

**Published:** 2019-10-11

**Authors:** Braedon McDonald, Mary Dunbar

**Affiliations:** ^1^Department of Critical Care Medicine, Snyder Institute for Chronic Diseases, Cumming School of Medicine, University of Calgary, Calgary, AB, Canada; ^2^Department of Pediatrics, Alberta Children's Hospital Research Institute, Cumming School of Medicine, University of Calgary, Calgary, AB, Canada

**Keywords:** platelets, sepsis, intravascular immunity, infection, thromboinflammation

## Abstract

Despite their humble origins as anuclear fragments of megakaryocytes, platelets have emerged as versatile mediators of thrombosis and immunity. The diverse spectrum of platelet functions are on full display during the host response to severe infection and sepsis, with platelets taking center-stage in the intravascular immune response to blood-borne pathogens. Platelets are endowed with a comprehensive armamentarium of pathogen detection systems that enable them to function as sentinels in the bloodstream for rapid identification of microbial invasion. Through both autonomous anti-microbial effector functions and collaborations with other innate immune cells, platelets orchestrate a complex intravascular immune defense system that protects against bacterial dissemination. As with any powerful immune defense system, dysregulation of platelet-mediated intravascular immunity can lead to profound collateral damage to host cells and tissues, resulting in sepsis-associated organ dysfunction. In this article, the cellular and molecular contributions of platelets to intravascular immune defenses in sepsis will be reviewed, including the roles of platelets in surveillance of the microcirculation and elicitation of protective anti-bacterial responses. Mechanisms of platelet-mediated thromboinflammatory organ dysfunction will be explored, with linkages to clinical biomarkers of platelet homeostasis that aid in the diagnosis and prognostication of human sepsis. Lastly, we discuss novel therapeutic opportunities that take advantage of our evolving understanding of platelets and intravascular immunity in severe infection.

## Introduction

Expanding beyond their well-known role in primary hemostasis, much of the recent research in the field of platelet biology has focused on elucidating the contributions of platelets to host defense and immunity. It is now well-established that platelets are integral to the innate immune response to infection and inflammation, both as autonomous effectors as well as collaborative conductors of anti-microbial defenses (1–6). The orchestral role of platelets in host defense is exemplified by their contribution to the intravascular immune response that unfolds during acute systemic infections, mediating host defense against microbial invaders while simultaneously contributing to organ dysfunction in sepsis (7). Below, we provide a comprehensive review of the cellular and molecular mechanisms of platelet functions in intravascular immunity as well as the pathogenesis of disease in sepsis. As detailed in the sections to follow, much has been learned from laboratory research and animal models of bacterial sepsis and endotoxemia, but it is helpful to begin with clinical observations from human sepsis that emphasize the critical importance of platelets in the pathogenesis of this systemic inflammatory disease.

## Platelets and Sepsis: The Clinical Perspective

Abnormalities of platelet homeostasis are common during acute infections and sepsis, and clinical monitoring of platelet counts has emerged as one of the most important biomarkers in the management of septic patients (8). The most commonly monitored platelet parameter is the peripheral blood platelet concentration, which is measured routinely (often daily) in hospitalized patients with acute infections. While quantitative assessments of platelets may seem rudimentary (and lacking information about functional properties), many clinical, and epidemiological studies have identified the peripheral blood platelet count as a useful diagnostic and prognostic biomarker in sepsis (9–22).

An acute rise in the circulating platelet count (acute thrombocytosis) is generally interpreted as a manifestation of systemic inflammation, as may be seen in the context of acute infection (23). However, the most common perturbation seen in acute infection and sepsis is a reduction in circulating platelets, and the development of acute thrombocytopenia (24). In fact, observational studies of the incidence and prevalence of thrombocytopenia in critically ill patients with sepsis and septic shock have reported that low platelet counts occur in 15–50% of patients (13, 16, 25, 26). Furthermore, among patients with normal platelet counts on admission, up to 44% will subsequently develop thrombocytopenia during the course of their stay in the intensive care unit (16, 21).

In addition to being a common finding in sepsis, clinicians have long appreciated that thrombocytopenia represents an ominous sign in the setting of severe infection (9–11, 14–20, 22). This clinical acumen is supported by data from multiple studies finding strong associations between low circulating platelet counts and adverse clinical outcomes in septic patients (9, 16, 19, 21, 22, 25, 26). Likewise, failure of platelet counts to recover into the normal range during acute illness is also associated with increased mortality, whereas recovery of platelet counts is strongly associated with survival to ICU discharge (14, 17, 18). The importance of this link between thrombocytopenia and sepsis pathogenesis is now solidified by the inclusion of thrombocytopenia as a core criterion for the diagnosis of sepsis. The updated consensus definition of sepsis uses the Sepsis-related Organ Dysfunction Score (SOFA) score, in which platelet count represents 1 of 6 core parameters (27). Impressively, severe thrombocytopenia carries the same prognostic significance in the SOFA score as major organ failure requiring life-support interventions (e.g., respiratory failure requiring mechanical ventilation, circulatory shock requiring vasopressors, severe renal and hepatic failure, or coma). Clearly, circulating platelet counts are an important biomarker of disease severity and clinical outcomes in sepsis.

The strong epidemiological links between abnormalities of platelet homeostasis and outcomes strongly support a role for platelets in the pathogenesis of sepsis and septic shock. The development of thrombocytopenia in sepsis occurs primarily as a result of massive consumption of circulating platelets through interactions with immune cells in the vasculature (described below), with additional contributions from reduced thrombopoiesis, sequestration, thrombotic microangiopathy (disseminated intravascular coagulation, DIC), direct pathogen-induced thrombocytopenia, immune-mediated thrombocytopenia, drug induced thrombocytopenia, and hemophagocytosis [see recent reviews by (8) and (28)]. Although thrombocytopenia is also seen in other causes of critical illness (trauma, burns, and others), it has been found that critical ill patients with burns develop more severe thrombocytopenia if there is concomitant infection and sepsis which is also linked to an increased risk of death, suggesting that sepsis-associated thrombocytopenia may have unique mechanisms of pathophysiology (29, 30). While much of our understanding of platelet functions in the immunopathogenesis of sepsis comes from elegant animal models of disease, a number of clinical studies have also shed light on the linkages between platelets and immunity in sepsis. Claushius et al. analyzed associations between admission platelet counts and immune phenotypes in 937 consecutive patients with sepsis admitted to 2 ICUs in the Netherlands, and found a strong association between severe thrombocytopenia, disease severity (APACHE IV score), and increased 1-year mortality (26). Using a propensity-matching strategy (to limit potential confounding by disease severity), the investigators found that, compared to patients with normal platelet counts, those with severe thrombocytopenia had higher levels of proinflammatory cytokines as well as increased markers of endothelial cell activation (increased sICAM-1 and fractalkine) and vascular permeability. Similar findings were reported in a cohort of critically-ill septic patients in Greece, in whom platelet counts varied inversely with serum pro-inflammatory cytokines and soluble ICAM-1 levels (31). Using whole blood transcriptomics, it was also shown that severe thrombocytopenia was associated with up-regulation of both pro- and anti-inflammatory signaling pathways, TLR signaling, and suppression of leukocyte adhesion molecule genes (26). Together, these findings indicate that platelet consumption and the development of thrombocytopenia is intimately associated with the dysregulated immune response that defines sepsis (27). In the following sections, the cellular and molecular mechanisms underlying these clinical observations will be reviewed in detail to explore the biological role of platelets in the intravascular immune response to infection, as well as the pathogenesis of sepsis.

## Platelets and Intravascular Immunity in Sepsis

### Platelets Are Immune Sentinels for Rapid Detection of Bloodstream Invaders

**Figure 1:**
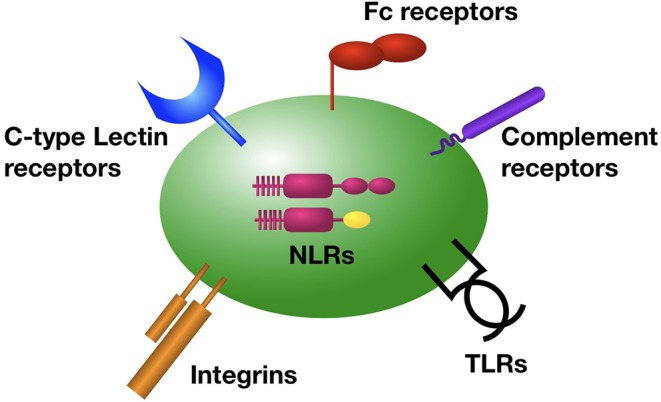
Platelet receptors for the detection of bacterial pathogens in the bloodstream. Platelets are equipped with a diverse array of surface and intracellular receptors for pathogen detection, enabling their function as intravascular sentinel cells. These include receptors for the detection of bacterial produces and molecules (TLRs, NLRs, C-type lectins, integrins, GP1bα), as well as bacteria that have been coated by antibody (Fc recpetors), complement (complement receptors), or von Willibrand factor (integrins and GP1bα).

Intravascular immune sentinels are strategically positioned within the vasculature to rapidly detect and respond to bloodstream invaders (32). Platelets play critical, yet underappreciated roles in the detection of pathogens in the bloodstream, and the elicitation of a coordinated immune response. Platelets are equipped with a comprehensive set of receptors that rapidly respond to invading pathogens and pathogen-associated molecular patterns (PAMPs) (Figure 1).

### Toll-Like Receptors

Like other immune sentinels, platelets express functional Toll-Like Receptors, including TLR 1, 2, 3, 4, 6, 7, 9 (33–40). However, activation of TLR signaling in platelets elicits a response that differs in many ways from other immune cells. First, platelets lack a nucleus, and therefore their response consists of an entirely post-transcription program. Platelet TLR signaling is incompletely understood, but appears to involve both MyD88-dependent and MyD88-independent signal transduction, including the activation of transcription factors such as NF-κB [recently review by (33, 41, 42)]. However, because platelets lack nuclei, these pathways lead to effector mechanisms that are independent of classical transcription programs, but the precise pathway details have not yet been fully elucidated [see (43) for a contemporary review]. Secondly, the activation thresholds for platelet TLRs have been found to differ from other immune cells in the context of sepsis and endotoxemia. The concentrations of LPS required to activate platelets via TLR4 *in vitro* is significantly higher than that required for activation of neutrophils (39). It has been hypothesized that this differential sensitivity to bacterial products allows platelets to reserve their arsenal for severe bloodstream infections with high concentrations of circulating PAMPs, thereby sparing the host from potential thromboinflammatory complications during minor infections (39). Thirdly, stimulation of platelets with LPS produces a functionally unique response compared to stimulation by other platelet activating substances (37, 39, 44). For example, Clark *et al*. found that stimulation of human platelets with classical activators like thrombin or PAF induced up-regulation of P-selectin, degranulation, and aggregation *in vitro*, while LPS did not (39). Instead, LPS induced a unique response linked to host defense, including adhesion to activated neutrophils and fibrinogen without marked aggregation or degranulation. Others have demonstrated a variety of additional effector functions elicited by TLR4 signaling in platelets, including the release of IL-1β and TNFα, tissue factor, and other immunostimulatory molecules, and augmented phagocytosis of platelets that are bound by autoantibodies (35, 36, 41, 45–47). Recently, it was shown that platelet TLRs can discriminate the nuances of specific PAMP characteristics, enabling tailored responses to different pathogens. Berthet et al. found that stimulation of platelets with structurally unique isoforms of LPS derived from *E. coli* or *Salmonella* yielded distinct responses (45). It was observed that incubation of PBMCs with supernatant from platelets stimulated with *S. enterica* LPS elicited significantly higher levels of IL-6, IL-8, and TNFα compared with supernatant from *E. coli* LPS-stimulated platelets. The mechanisms that enable platelet TLR4 signaling to discriminate between pathogen-specific LPS isoforms remains unknown, but these observations indicate that platelet sentinels possess the sensitivity to differentiate between microbes and induce tailored responses. Taken together, the versatile functions of platelet TLRs exemplify the sentinel characteristics of platelets in the bloodstream; poised to rapidly but precisely identify invading pathogens and initiate an appropriate and tailored response.

### Other Pathogen Detection Mechanisms

In addition to TLRs, platelets are equipped with a variety of other receptors to detect pathogen invasion (Figure 1). Platelets have been shown to express functional intracellular pattern-recognition receptors (PRRs) including NOD2 and NLRP3, but their physiologic function in platelets remain to be defined (48, 49). Platelets can also recognize and respond to bacteria that have been opsonized by humoral mediators of innate and adaptive immunity (50). Fixation of C3b to the surface of bacteria can be recognized by platelets in a GP1b-dependent manner, resulting in the formation of circulating platelet-bacteria complexes that facilitate delivery of bacteria to professional phagocytes (51). Human platelets also express a range of functional immunoglobulin Fc receptors for IgG, IgA, and IgE to detect antibody-laden bacteria (50). Platelet GPIIbIIIa (αIIbβ3) and GP1bα can also facilitate binding to bacteria, either directly to bacterial surface proteins or via molecular bridges provided by fibrinogen, fibronectin, or von Willibrand factor (52). Platelets express C-type lectin receptors CLEC-2 and DC-SIGN that have been shown to mediate binding to viral particles, and also contribute immunomodulatory effects during bacterial sepsis (53, 54). Lastly, collaboration between multiple receptor types may be required for platelets to detect and respond pathogens. For example, the response of human platelets to both Gram-positive and Gram-negative bacteria was shown to be dependent on FcγRIIA activation by IgG-bound bacteria, but only with concomitant engagement of GPIIbIIIa (αIIbβ3) (55, 56).

In addition to direct sensing of bacteria and bacterial products, platelets can become activated in response to inflammatory mediators liberated by other sentinels during acute infection. Platelets are decorated with a number of cytokine and chemokine receptors that detect prototypical signals of acute inflammation (57). Furthermore, platelets are potently activated by a variety of damage-associated molecular patterns (DAMPs) that are released from stressed and dying cells during acute infection. Interestingly, intravenous administration of purified danger signals such as histone proteins induces profound platelet activation and thrombocytopenia similar to that seen during endotoxemia and sepsis (58). Therefore, platelets are endowed with the machinery to detect and respond to both primary (bacteria and bacterial products) and secondary (DAMPs and inflammatory mediators) signals of acute infection, providing the necessary redundancy to function as effective sentinels within the bloodstream.

Lastly, in addition to their ability to detect passively circulating signals of pathogen invasion, platelets also conduct active surveillance of the microcirculation to enable a rapid and focused response to endovascular pathogens. Intravital imaging analysis of platelet behavior within the microcirculation of highly vascular organs (lungs, liver, brain) has revealed that platelets undergo transient touch-and-go interactions with the vascular endothelium and other intravascular immune cells such as Kupffer cells in the liver (59). Under homeostatic conditions, platelets were observed to instantaneously touch-down in a GP1b-dependent manner, and in the absence of bacteria would immediately release and return to the circulation (59). When pathogenic Gram-positive bacteria such as *Bacillus cereus* or *Staphylococcus aureus* were introduced into the bloodstream and captured by liver Kupffer cells, these touch-and-go interactions converted to adhesion and aggregation nucleated around the captured bacteria. Mice deficient in GP1bα, which were incapable of touch-and-go interactions, rapidly succumbed to overwhelming infection, demonstrating that platelet surveillance is essential for effective host defense against blood-borne bacteria (59). It remains unknown whether this active microvascular surveillance is required for control of other types of bloodstream infections (Gram negative, fungal, parasitic), and how this behavior is regulated to avoid overwhelming microvascular thrombosis. In addition, further research is required to define the molecular events that enable platelets to convert from surveillance to aggregation upon pathogen encounter, and whether dysregulation of this behavior contributes to disease pathogenesis in sepsis and other thromboinflammatory disorders.

### Platelets and Microvascular Traffic Control in Sepsis

The early phase of sepsis is characterized by a vigorous systemic inflammatory response, during which platelets (together with leukocytes, primarily neutrophils) are recruited from the circulation and sequestered within highly vascular organs such as the lungs and liver, resulting in consumptive thrombocytopenia (37, 39, 60–65). Interestingly, although the spleen is a reservoir for large numbers of platelets, studies that have tracked radio-labeled platelets in the circulation of septic mice have found little contribution of the spleen to the development of thrombocytopenia in sepsis (37, 65). Once considered a non-specific and maladaptive reaction to severe infection, contemporary evidence suggests that the recruitment of platelets into the microcirculation of the liver and lungs is part of a highly coordinated intravascular immune response involving collaboration between platelets and leukocytes (7). Platelets have emerged as central regulators of the intravascular immune response, beginning with the orchestration of immune cell trafficking within the inflamed microvasculature. In this section, we will discuss the role of platelets in the coordination of immune cell recruitment to inflamed tissues during infection and inflammation, followed in the next section by a review of the role of platelets in regulating antibacterial functions of immune effector cells.

Platelets engage in a reciprocal relationship with neutrophils to coordinate their recruitment and function within the microvasculature. A number of studies have reported that depletion of neutrophils or inhibition of neutrophil recruitment prevents platelet consumption and the development of thrombocytopenia, suggesting that neutrophils are essential for platelet sequestration within the microcirculation (39, 62). Indeed, direct visualization of platelet-neutrophil dynamics in the pulmonary and hepatic microcirculation using intravital microscopy has shown that neutrophil adhesion is followed immediately by platelet binding and aggregation upon their surface (62, 66–68). Similar mechanisms of neutrophil-dependent platelet recruitment have been observed in a number of organ systems, and across a variety of sepsis models (37, 39, 62, 64, 66). A number of adhesion mechanisms can support platelet binding to neutrophils under flow conditions. First, selectin-mediated interactions can support adhesion between platelets and neutrophils *in vitro* and *in vivo* via both direct and indirect mechanisms. Interactions between selectins and selectin-ligands are best known for mediating transient low-affinity catch-bonds that support leukocyte rolling on activated endothelium (69, 70). In contrast, binding between platelet P-selectin and neutrophil PSGL-1 can mediate stable adhesion between these cells (61, 68, 71, 72). Given the low-affinity nature of their binding, it is likely that P-selectin—PSGL1 interactions between platelets and neutrophils are particularly effective within the low-shear environment of the pulmonary and hepatic capillaries. Furthermore, PSGL1 engagement can amplify platelet-neutrophil adhesion by stimulating “outside-in” signaling pathways in neutrophils (including Src-family kinase and MAP kinase pathways) that induce the activation of integrins (73–76). Neutrophil Mac-1 (α_M_β_2_ integrin) can mediate adhesion to platelets via multiple receptors, including GP1bα as well as GPIIbIIIa via a fibrinogen bridge (77, 78). Alternatively, it has been shown that binding between human neutrophils and platelets in response to plasma from septic patients can be mediated by engagement of LFA-1 (α_L_β_2_ integrin) and ICAM-2 (62).

The multitude of adhesion mechanisms that support platelet-neutrophil binding reflects the complexity of their interactions *in vivo*. Studies using intravital microscopy have revealed that adherent neutrophils nucleate the formation of large, dynamic aggregates that fluctuate in size over time as a result of continuous binding and release of circulating platelets (62, 66, 67). Furthermore, these dynamic platelet aggregates migrate throughout the vasculature atop neutrophils as they crawl along the endothelial surface (62, 66, 67). The molecular mechanisms that regulate this dynamic aggregation and the functional role of continuous platelet recycling atop neutrophils are unclear, but are likely tightly controlled to avoid catastrophic microvascular thrombosis.

Under certain inflammatory contexts (primarily non-infectious), the recruitment of platelets and neutrophils within the microcirculation may follow the opposite sequence, that is, initial platelet accumulation upon the endothelium followed by subsequent neutrophil recruitment. This mode of platelet-dependent neutrophil recruitment has been observed in a number of models of inflammatory and thrombotic disease, including cytokine-induce cerebral inflammation (79), thermal liver injury (80), acute lung injury (61, 81), and venous thrombosis (82). In these contexts, platelets adhere and aggregate at sites of compromised vascular integrity, and then promote neutrophil recruitment either directly through P-selectin-PSGL-1 and/or integrin-mediated interactions, or indirectly through TXA1-mediated activation of endothelium (83). In instances where vascular integrity is severely disrupted, platelets can even be seen “paving” neo-vessel-like conduits through which neutrophils migrate to reach sites of inflammation (80). Thus, the trafficking and recruitment of platelets and neutrophils exists as a reciprocal relationship, in which neutrophils may recruit platelets, and platelets may recruit neutrophils. The mechanisms that dictate neutrophil-first vs. platelet-first recruitment are not well-understood. It is possible that the nature of the inflammatory stimulus (e.g., infectious vs. non-infectious), or the microvascular characteristics (endothelial integrity, adhesion molecule expression) may induce different patterns of cell recruitment to tailor the intravascular immune response to the inciting stimulus. As described in the sections below, this hypothesis is supported by evidence that neutrophil effector functions can differ in response to platelet-first compared to neutrophil-first recruitment interactions (80).

Following adhesion to the endothelium, immune cells migrate through the vasculature guided by a variety of chemotactic cues (84–86). The platelet payload includes a number of chemoattractant factors that can guide the chemotaxis of neutrophils and other leukocytes (4). In addition, platelets may directly influence neutrophil chemotaxis within blood vessels through contact-mediated interactions on specific microdomains of the neutrophil surface. Sreeramkumar et al. conducted a detailed *in vivo* investigation of neutrophil polarization (an essential pre-requisite for directional migration), and made the striking discovery that platelets dock to the leading-edge of adherent neutrophils in the vasculature, and that this polarized binding was crucial for directional migration (68). Following engagement of platelet P-selectin to PSGL-1 on the leading edge of neutrophils, outside-in signal transduction led to a redistribution of surface receptors Mac-1 (α_M_β_2_ integrin) and CXCL2, generating polarized receptor microdomains that were essential for effective locomotion toward infection and injury. This discovery revealed that adherent neutrophils scan for activated platelets in the vasculature to enable physical interactions that steer neutrophil migration toward appropriate targets.

Lastly, like other innate immune cells, platelets themselves possess a rudimentary ability to migrate (undergo chemotaxis) in response to chemoattractant stimuli. Platelets express a variety of surface receptors for prototypical chemoattractants, as well as the necessary intracellular signal transduction and cytoskeletal machinery required for cell motility. Platelet chemotaxis remained an *in vitro* observation for many years (4), until a number of recent studies demonstrated evidence of platelet migration *in vivo* in mouse models of allergic pneumonitis and sepsis (87, 88). Directional migration of platelets *in vivo* relies on integrin-based interactions, as blockade of GPIIbIIIa (αIIbβ3) integrin inhibits intravascular platelet locomotion (88). The physiologic function of platelet locomotion remained a biological curiosity until recently, when Massberg et al. published a seminal study detailing a functional contribution of platelet migration to anti-bacterial host-defense *in vivo* (88). The authors observed that migrating platelets behaved as “mechano-scavengers” within the vasculature, piling-up substratum and other particles (including bacteria) as they moved. This mechano-scavenger behavior enabled platelets to collect bacteria that they encountered in their travels, bundling them within open cannalicular systems (OCS) for disposal by professional phagocytes. Therefore, migration of platelets appears to fill an important niche in the intravascular immune response by contributing to the collection and clearance of pathogens in collaboration with local phagocytes.

### Platelets and Neutrophils: Partners in Intravascular Immunity During Sepsis

Platelets have emerged as versatile effectors of anti-bacterial immunity, contributing both autonomous bacteriocidal/bacteriostatic properties as well as synergistic partnerships with other innate immune cells. In co-culture experiments, platelets can autonomously inhibit the growth of various bacteria (89–91). The anti-bacterial properties of platelets have largely been attributed to their ability to produce anti-microbial peptides such as β-defensins and so-called “platelet-microbicidal proteins” (PmP) [reviewed by (50)]. In addition, it has been observed that platelets exhibit a rudimentary ability to engulf bacteria (92). However, the functional significance of these autonomous anti-bacterial properties in the context of *in vivo* host defense remains unknown.

**Figure 2:**
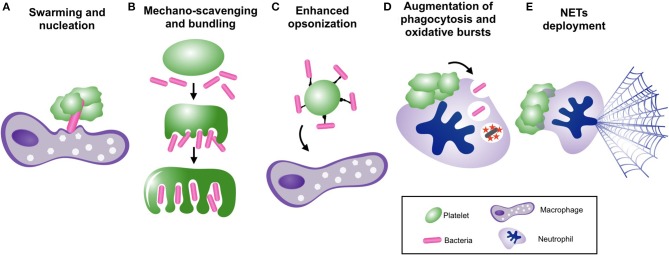
Platelets collaborate with neutrophils and other immune cells to facilitate bacterial clearance from the bloodstream. Mechanisms of bacterial clearance facilitated by platelet-leukocyte interactions, including, **(A)** vascular surveillance and nucleation of bacterial invaders to promote phagocytosis by macrophages, **(B)** mechano-scavenging of bacteria within the microvasculature, **(C)** “opsonization” of circulating bacteria for clearance by phagocytes **(D)** augmentation of neutrophil-mediated phagocytosis and oxidative killing, and **(E)** induction of neutrophil extracellular trap (NET) release.

In contrast, platelets have well-defined anti-bacterial functions *in vivo* that arise from synergistic partnerships with other immune cells (Figure 2). For example, platelets are crucial for survival in mouse models of *Bacillus cereus* and *Staphylococcus aureus* bacteremia due to their ability to collaborate with liver macrophages to clear circulating bacteria (59). During these infections, platelets were observed to aggregate upon bacteria-laden macrophages in the liver, providing an essential signal to macrophages that enabled clearance of bacteria from the circulation and protection against overwhelming sepsis (59). In addition, circulating platelets can scavenge blood-borne bacteria and enhance their delivery to phagocytes (51). However, it should be noted that under certain circumstances, platelet-mediated bacterial “opsonization” may actually impair clearance of bacteremia by diverting microbes toward less-efficient phagocytes, as is seen in *Listeria monocytogenes* infection (51).

The most extensively studied and perhaps most potent platelet-mediated anti-bacterial responses are generated through collaboration between platelets and neutrophils. Platelet-neutrophil interactions induce and/or augment a number of anti-bacterial neutrophil functions to enhance the clearance of bacteria from the bloodstream. First, neutrophil phagocytosis of extracellular bacteria is augmented by mediators released from activated platelets (93, 94). In addition, platelets augment intracellular killing of bacteria within neutrophils by promoting oxidative burst generation through both contact-dependent outside-in signaling as well the release of various soluble mediators (76, 95, 96). In addition to augmenting phagocytosis and intracellular killing, platelets help supply neutrophils with their prey through mechano-scavenging behavior in the microvasculature, bundling stray bacteria for efficient phagocytosis and clearance by neutrophils (and other phagocytes) (88).

Lastly, the most potent anti-microbial effector mechanism unleashed in response to platelet-neutrophil interactions is the neutrophil extracellular trap (NET). NETs are extracellular webs of decondensed chromatin laden with proteolytic enzymes and other anti-bacterial molecules that are expelled from activated neutrophils (97). NETs are capable of both capturing and directly killing extracellular microbes, including bacteria, fungi, parasites, and even display anti-viral properties (98). Although a number of activating stimuli can induce NETs release from neutrophils, activated platelets have emerged as one of the most potent stimuli for NETs release, and platelet-induced NETs have been observed in a variety of sepsis models as well as other non-infectious inflammatory conditions (39, 62, 63, 82, 99–101). During sepsis, neutrophils cast NETs into the bloodstream to filter pathogens from the circulation (39, 62, 63). This powerful and efficient bacterial clearance system is strategically positioned within the microcirculation of highly vascular organs (liver and lung), enabling maximal filtration of the cardiac output to protect against hematogenous dissemination of infection (62).

The molecular mechanisms controlling NETs release in response to platelet-neutrophil interactions are incompletely understood. Blocking the physical engagement of platelets with neutrophils inhibits the release of NETs, suggesting a contact-dependent induction pathway. However, while contact between neutrophils and platelets is essential for intravascular NETs release *in vivo*, it is not sufficient. Using a mouse model of sterile pulmonary inflammation, Rossaint et al. found that a second signal composed of platelet-derived CXCL4/CCL5 heterodimers was essential for NETs release in response to platelet-neutrophil binding (100). *In vitro*, β-defensin-1 released from activated human platelets was shown to induce NETs release from neutrophils, but this is yet to be confirmed *in vivo* (89). A full understanding of the molecular mechanisms of platelet-mediated NETs release during bacterial sepsis *in vivo* remains to be elucidated.

Finally, although platelet-neutrophil collaboration has been studied more extensively, it should be noted that platelets engage in functional interactions with other innate immune cells in the vasculature during sepsis. Platelet-monocyte complex formation has been observed in the blood of septic patients (102, 103), and the levels of circulating platelet-monocyte complexes may be a useful biomarker to predict adverse outcomes in older adults with sepsis (102). Functionally, platelets have been shown to aid in the recruitment of monocytes to foci of Listeria monocytogenes infection in mice, as well as modulate the inflammatory phenotype of monocytes during viral infection (104, 105). Platelets have also been shown to modulate the polarization, cytokine profile, and antibacterial effector mechanisms of macrophages in animal models of sepsis (59, 91, 106, 107).

Overall, there is strong evidence that collaboration between platelets, neutrophils, and other antibacterial effector cells within the microvasculature is crucial to protect against blood-borne infections. Unfortunately, as with most powerful immune defense mechanisms, dysregulation of the intravascular immune response during sepsis can result in tremendous collateral damage to host cells and tissue, resulting in organ dysfunction.

### Platelets-Neutrophil-NETs Axis and Organ Dysfunction in Sepsis

**Figure 3:**
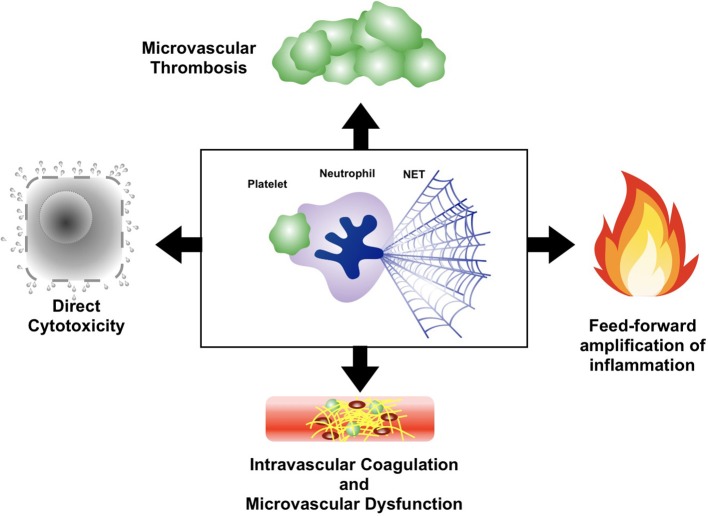
Mechanisms of organ dysfunction in sepsis induced by the platelet-neutrophil-NETs axis. Intravascular collaboration between platelets, neutrophils, and NETs leads to cell and tissue damage as a result of directly cytotoxicity, induction of intravascular coagulation and microvascular dysfunction, and propagation of a dysfunctional thromboinflammatory response through feed-forward microvascular inflammation and thrombosis.

The intricate interplay between platelets, neutrophils, and NETs in sepsis represents a classical example of a “double-edged sword”; providing protective host defense while simultaneously causing immune-mediated organ dysfunction (Figure 3). The importance of this axis in sepsis pathogenesis has been confirmed in a variety of animal models of acute infection, in which therapeutic blockade of the platelet-neutrophil-NETs axis reduced organ dysfunction and (in some models) improved survival (62, 67, 108–115). Platelets and neutrophils conspire together to induce microvascular dysfunction and tissue damage through a variety of mechanisms. In particular, the release of NETs in response to platelet-neutrophil engagement can be especially cytotoxic to host cells. During sepsis, NETs can be found throughout the vast microvasculature of the liver and other organs, exposing the endothelium and underlying parenchyma to a variety of potent cytotoxic mediators. First, extracellular histones proteins contained within the chromatin backbone of NETs are particularly cytotoxic to endothelial cells and parenchymal cells *in vitro* and *in vivo* (116). In fact, Esmon et al. revealed that antibody-mediated neutralization of histone proteins in a mouse model of polymicrobial sepsis protected against multi-organ dysfunction and death (116). Furthermore, NETs contain an abundance of proteolytic and antimicrobial proteins that can damage host cells and tissues. For example, genetic deficiency of neutrophil serine proteases such as neutrophil elastase, or blockade of their enzymatic activity with small molecule inhibitors, resulted in a dramatic decrease in biomarkers of tissue damage in mouse models of acute infection (115). Lastly, the fact that many NETs components can serve as pro-inflammatory DAMPs (DNA, histone proteins, proteolytic enzymes, antimicrobial peptides, and others) creates in a feed-forward system that propagates microvascular inflammation and amplifies tissue damage.

In addition to direct cytotoxicity, a growing body of literature implicates the platelet-neutrophil-NETs axis in sepsis pathogenesis through the induction of intravascular coagulation. Platelet-neutrophil interactions and the subsequent release of NETs coincides with diffuse activation of thrombin and fibrin deposition within the vasculature of multiple organs (67). Imaging of this process *in vivo* has revealed disseminated microvascular coagulation following NETs release, resulting in extensive obstruction of blood flow and ischemic injury to the affected organ (67). This widespread microvascular coagulation was largely NETs dependent, as mutant mice with severely reduced NETs production (peptidylarginine deiminase 4 [PAD4] deficient mice) have markedly diminished intravascular coagulation and organ injury, yet still have abundant neutrophil-platelet aggregation in the microvasculature (67). Conversely, studies using DNase-deficient mice have revealed that impaired clearance of NETs (leading to an overabundance of NETs in the vasculature) precipitates extensive microvascular coagulation and thrombosis. In fact, administration of endotoxin to DNase1-deficient or DNase-like-3 deficient mice resulted in multi-organ microvascular coagulation and microangiopathic hemolytic anemia characteristic of disseminated intravascular coagulation (DIC), followed by death within hours (112). There is also emerging evidence from human studies that NETs promote hypercoagulability in patients with sepsis, and that elevated levels of circulating NETs is associated with sepsis-related disseminated intravascular coagulation (117–119).

The pro-coagulant properties of NETs arise from a variety of components that are known to interact with the clotting cascade at multiple levels. For example, histone proteins have been shown to initiate coagulation through the upregulation of tissue factor as well as initiation of the contact-dependent pathway (63, 82, 120). Indeed, antibody-mediated neutralization of histone H4 within NETs in a mouse model of Gram-negative sepsis reduced intravascular thrombin generation in the liver and lung microcirculation (67). Furthermore, neutrophil serine proteases present within NETs (such as neutrophil elastase and cathepsin G) activate tissue factor- and factor XII-dependent coagulation pathways, and also promote platelet activation through protease-activated receptors (PARs) (63, 121). NETs may also acquire pro-coagulant factors from the bloodstream to bolster their ability to initiate and propagate microvascular coagulation. Circulating microparticles are captured by NETs that can augment thrombin generation via factor XII and the intrinsic pathway (122). Lastly, cross-talk between intravascular NETs and endothelial cells leads to upregulation of tissue factor expression and direct activation of the coagulation cascade within the vessel lumen (123).

Although a number of components within NETs have the ability to activate coagulation in isolation, emerging evidence suggests that the induction of intravascular coagulation *in vivo* requires the intact NETs macrostructure. Most notably, experiments using exogenous DNase to dissolve the DNA backbone of NETs have demonstrated marked inhibition of intravascular thrombin activation and fibrin production, despite the fact that individual pro-coagulant NETs components remain in the vasculature (67, 122). These findings suggest that intact NETs provide an essential catalytic scaffold for the induction of disseminated intravascular coagulation during sepsis.

Finally, in addition to stimulating NETs release from neutrophils, platelets also provide synergistic amplification of NET-induced coagulation. While the majority of platelets are sequestered in the microvasculature through interactions with neutrophils, platelets can also bind and aggregate directly on NETs. NETs-mediated platelet aggregation has been demonstrated in a variety of thromboinflammatory disorders, including sepsis, venous thromboembolic disease, and atherosclerotic vascular disease (67, 124, 125). During sepsis, platelets aggregate within intravascular NETs and amplify thrombin generation. The molecular crosstalk between platelets and NETs that propagates intravascular coagulation is incompletely understood, but one study identified platelet polyphosphate (released from dense granules) as an essential signal for NETs-mediated thrombin activation *in vivo* (67), and polyphosphate has been identified as a cofactor for histone H4-mediated thrombin activation *in vitro* (120). Overall, the extensive cross talk between platelets, neutrophils, and NETs results in widespread intravascular coagulation, microvascular occlusion, and ischemic and cytotoxic organ damage that is characteristic of sepsis pathology.

## Conclusions

Platelets are versatile mediators of antimicrobial immunity and host defense within the bloodstream during sepsis (Table 1). However, dysregulation of this platelet-mediated intravascular immune response leads to tissue damage, intravascular coagulation, and organ dysfunction. The pathological integration of immunity, thrombosis, and coagulation in sepsis provides a glimpse into why clinical trials of anti-inflammatory or anti-coagulant therapies alone have yielded underwhelming results, and underscores the importance of creating novel therapies that target keystone mechanisms in this complex pathological system. The integrated response of platelets, neutrophils, and NETs represents one such keystone mechanism, with a growing body of literature demonstrating therapeutic efficacy of targeting this system in animal models of sepsis and other acute inflammatory diseases. In humans, retrospective studies of patients with septic shock have found that anti-platelet agents may reduce the risk of end-organ dysfunction and even mortality (128–130). Furthermore, the upcoming AspiriN To Inhibit SEPSIS (ANTISEPSIS) trial will investigate the use of low-dose aspirin as a primary preventative measure to reduce sepsis-related mortality and organ dysfunction in elderly patients (131). Beyond prototypical anti-platelet agents, other novel therapeutic targets have emerged from our understanding of the cellular and molecular mechanisms of platelet function in sepsis. For example, an abundance of preclinical data described above supports the use of therapies targeting the platelet-neutrophil-NETs axis to protect against microvascular dysfunction, organ damage, and death in models of bacterial sepsis and endotoxemia. With a growing list of direct and indirect inhibitors of NETs production and/or function, clinical trials of NETs-blockade in sepsis are on the horizon. Of course, as with any immunomodulatory therapy, blockade of the platelet-neutrophil-NETs axis may produce unwanted side effects including defects in host defense. Therefore, continued research is needed to generate optimized treatment strategies that functionally uncouple the harmful pathogenic mechanisms from the protective immune properties of this intravascular immune response.

**Table 1:** The role of platelets in host defense during bacterial infection in the bloodstream.

**Host response in sepsis**	**Roles of platelets**	**Selected references**
Pathogen detection	Direct detection of invading pathogens (see Figure 1)	(34, 35, 37, 39)
	Intravascular surveillance behavior	(59, 88)
Pathogen capture and killing	Direct platelet-mediated cytotoxicity	(89–91)
	Enhanced opsonization	(36, 51, 53, 59)
	Enhancement of leukocyte-mediated capture and killing	(51, 59, 62, 88, 93, 94)
	Induction of intravascular NETs	(39, 62, 63, 99)
Modulation of immune cell trafficking and function	Platelet trafficking and sequestration in microcirculations	(37, 39, 60–63, 65, 67)
	Neutrophil trafficking and function	(61, 68, 79, 81)
	Monocyte trafficking and function	(102–105)
	Macrophage polarization and function	(59, 91, 106, 107)
Modulation of systemic inflammatory response	Inflammatory mediator release from platelets	(36, 46, 47)
	Inflammatory-mediator production by innate immune cells	(26, 45, 106)
	Complement activation and anaphylatoxin production	(126, 127)
Microvascular thrombosis and coagulation	Activation and propagation of intravascular coagulation	(67, 112)
	Sepsis-associated DIC	(112, 117–119)

## Author Contributions

BM performed the literature review, wrote and edited the manuscript. MD contributed to the literature review, manuscript preparation, and figure development.

### Conflict of Interest

The authors declare that the research was conducted in the absence of any commercial or financial relationships that could be construed as a potential conflict of interest. The handling Editor declared a shared affiliation, though no other collaboration, with the authors at time of review.
